# Effectiveness of Systemic Inflammation Response Index (SIRI) Neutrophil–Lymphocyte Ratio (NLR), Derived Neutrophil–Lymphocyte Ratio (dNLR), and Systemic Immune Inflammation Index (SII) for predicting prognosis of acute diverticulitis

**DOI:** 10.1007/s13304-025-02241-x

**Published:** 2025-05-15

**Authors:** Yasin Alper Yıldız

**Affiliations:** Department of General Surgery, Faculty of Medicine 37120, Kastamonu University, Kastamonu, Turkey

**Keywords:** Colonic Diverticulitis, Prognosis, Neutrophil, Inflammation

## Abstract

There are many applications to emergency services due to acute colonic diverticulitis. It is necessary to decide whether these patients are complicated, to quickly predict their prognosis, and to decide whether medical or invasive treatment is necessary. We planned to research effectiveness NLR, NLR, SII, and SIRI values calculated using hemogram data can predict the prognosis of acute diverticulitis. We managed a retrospective scanning with patients who applied with a diagnosis of acute diverticulitis between 06/2020 and 04/2023. Demographic data (age, gender, previous surgery, comorbid diseases), tomographic Hinchey classification, location of diverticulitis, applied treatment, and obtained from blood parameters at presentation to the emergency department WBC, CRP, NLR, dNLR, SII, SIRI parameters were recorded from the electronically registered patient files. According to the Hinchey classification, those with Hinchey 0 and 1a were included in the noncomplicated group A class because they were followed up with outpatient medical follow-up. Those with Hinchey 1b,2,3,4 were included in the complicated GROUP B class. Differences in WBC, CRP, NLR, dNLR, SIRI, SII values between the complicated and noncomplicated groups were evaluated statistically. There were 286 patients with acute diverticulitis on the dates indicated. The number of patients eligible for the study was eighty-two. According to Hinchey’s classification, 56 patients had noncomplicated acute diverticulitis (SAD) and 26 patients had complicated diverticulitis (CAD). These diagnoses were given based on tomography findings and clinical evaluations. The hospital stay was longer in the CAD group compared to the SAD group (p < 0.001) statistically significantly. The rate of surgical procedures and percutaneous interventions in the CADs was higher than SADs (p: 0.040) statistically significantly. WBC (white blood cell), NLR, dNLR, SIRI, SII and CRP parameters were higher in CADs than in SADs as statistically significant. Spearman’s correlation analysis showed between the Hinchey classification and the NLR, dNLR, SII, SIRI, CRP, WBC parameters with high correlation as positive. Determination of values SIRI (2.42), NLR (3.35), SII (907.44) dNLR (4.63), CRP (15.25) WBC (11.16) and specificity and fractionation of these values ROC analysis was performed for this purpose. Highest AUC (area under the curve) value was found in WBC [0.807 0.703–0.910)], SIRI [0.786 (, 0.681–0.892)], SII [0.767 (0.654–0.880)], NLR [0.740 (0.624–0.854)], dNLR [0.739 (0.625–0.853)]. This study showed that there are SII, SIRI dNLR, NLR, CRP, and WBC values in patients presenting with acute diverticulitis a very high correlation with Hinchey classification in distinguishing complicated and non-complicated acute diverticulitis (*p* < 0.01). These data were higher in CADs than in SADs statistically significantly. The use of these data can both prevent unnecessary radiation in patients suspected of acute diverticulitis by reducing unnecessary tomography scans and can be valuable in predicting the prognosis of diverticulitis at a low cost.

## Introduction

Diverticulosis occurs when mucosal protrusions form due to deformities in the bowel wall [1]. The herniation of the mucosa in the colon wall and the formation of diverticula are due to the increase in intraluminal pressure at weak points [2]. Various complications can be seen such as acute uncomplicated diverticulitis, painless bleeding, perforation and complicated diverticulitis in diverticulosis. Uncomplicated diverticulitis is seen in 1–25% of patients with diverticulosis [3, 4]. Phlegmon, abscess, and perforation as complication in diverticulosis were evaluated according to the Hinchey classification defined by Hinchey et al. [5]. The Hinchey classification was modified by Wasvary et al. in 1999 [6] and Kaiser et al. in 2005. Second one is especially important because it includes computed tomography (CT) findings [7] (Table 1).

**Table 1:** Classification modified by Wasvary et al. (1999) and by Kaiser et al. (2005)

Hinchey sınıflaması	Classification modified by Wasvary et al^6^	Classification modified by Kaiser et al.(CT findings)^7^
0	Colonic wall thickening	Colonic wall thickening
1a	Phlegmon: confined pericolic inflammation	Same findings as 0 + pericolic tissue changes
1b	Pericolic or mesocolic abscess	Same findings as 1a pericolic or mesocolic abscess
2	Pelvic, abdominal or retroperitoneal abscess(distant abscess)	Same findings as 1a + distant abscess
3	Generalized purulent peritonitis	Pneumoperitoneum associated with localized free fluid or ascites and possible wall thickening
4	Generealized fecal peritonitis	Same findings as 3

Abdominal tenderness, and greater C-reactive protein (CRP) levels than 5 mg/dl and vomiting is important for detecting acute diverticulitis(AD). The distinction between complicated and uncomplicated diverticulitis cannot be made based on this triad. [8]. An evaluation that includes history, inflammatory markers, physical examination findings and radiological findings is advised. Abdominal tomography may be performed in possible acute diverticulitis patients. [9]. Such patient management may lead to unnecessary CT scans in uncomplicated diverticulitis patients. Biomarkers are used as noninvasive, reliable and inexpensive tools that can support the diagnosis of CAD in diverticulitis like other diseases. For example, CRP is an effective marker for estimating the level and severity of AD’s inflammation [10]. Zahorec defined NLR as a marker of inflammation [11]. Platelet/lymphocyte ratio (PLR) has been used in inflammatory diseases, infectious and malignant patients as a prognostic factor [12]. NLR, PLR have been shown to be preferable markers for the assessment of infectious intensity compared to CRP, WBC and absolute neutrophil [13]. Systemic immune-inflammatory index (SII) has been accepted as an important marker of inflammation and immune response [14, 15]. NLR, PLR, SIRI and SII can show immune response and systemic inflammation status [16].

Tomographic data of acute diverticulitis patients were examined and according to the Hinchey classification, those with Hinchey 0 and 1a were classified as uncomplicated (simple) acute diverticulitis (SAD), Hinchey 1b and others were classified as complicated acute diverticulitis (CAD) because they require closer follow-up and treatment in our study. Prognosis in patients with complicated diverticulitis was determined by looking at the NLR, MLR, dNLR, PLR, SII, and SIRI values in these patients. There are some studies that have used these biochemical markers in different diseases in the literature, but NLR and PLR values have been studied in some publications in diverticulitis patients and only two studies have evaluated them with SII [17, 18] and no diverticulitis studies have been conducted with SIRI and dNLR. In this respect, our study is unique in literature and will contribute to literature.

## Patients/methods

We planned a retrospective study of acute diverticulitis patients who were admitted to the hospital between 06/2020 and 04/2023. Patients were screened according to their ICD-9 codes, and their epicrisis, radiological imaging, and hemogram and biochemistry markers were extracted. Patients who applied for acute diverticulitis were included over the age of 18. Patients who have a pathology other than acute diverticulitis, whose data were incomplete, and who were on immunosuppressive therapy were excluded from the study.

Comorbid diseases, tomographic Hinchey classification, location of diverticulitis, laboratory data (hemogram data) from patient files recorded electronically in the hospital system. NLR, SII, and SIRI values were calculated. NLR = (neutrophil:lymphocyte); PLR = (platelet:lymphocyte); SII = (neutrophil x platelet):lymphocyte count and SIRI = (neutrophil x monocyte):lymphocyte [19], dNLR = (white blood cell-lymphocyte):lymphocyte [20].

Patients with Hinchey 0 and 1a were included in the noncomplicated group A class because they were followed up with outpatient medical follow-up. Hinchey 1b, 2, 3, and 4 patients were in the complicated group B class. Medical, interventional or surgical treatments and hospital stays of these patients were recorded.

## Statistical analysis

We used an independent *t*-test for normally distributed data. Chi-square test was used for variables between two independent groups. We used Mann–Whitney U test for numerical data. It was considered statistically significant for *p* < 0.05. MLR, PLR, NLR, dNLR, SIRI and SII were investigated area under the curve (AUC). For this, we use receiver-operating characteristic curve analysis (ROC) to establish the association of the PLR, MLR, dNLR, NLR SIRI, SII with tomographic Hinchey classification. We established cut-off points with the Youden index. We determined cut-off points at appropriate specificity and sensitivity ratios that correlate these values with the advanced Hinchey classification. We used SPSS Version 22 for a statistical analysis (IBM, Armonk, NY, USA).

## Results

There were 286 patients diagnosed with acute diverticulitis, 82 of whom had the inclusion criteria.

Forty-one of the patients (50%) were female and Fourty-one of the patients (50%) were male (Table 2). Mean age of patients was 60.5 (23–101).

**Table 2:** Differences in time of hospital stay surgical procedure requirement, diverticulum location and demographic data between the two groups

	SAD(simple acute diverticulitis)	CAD(complicated acute diverticulitis	*p*
Number of patients	56(68.3%)	26(31.7%)	
Median age(Years)	63.5(23–85)	57(34–101)	> 0.05
Days of stay in hospital	2.44(0–10)	5.30(0–30)	0.000
Gender			> 0.05
Female	29	12	
Male	27	14	
Surgical/medical treatment			0.04
Surgical	0	4	
Percutaneous intervention	0	1	
Medical	56	21	
Location			> 0.05
Ascending colon	6	3	
Desceived and sigmoid colon	46	23	
Ascending colon + left colon	2	0	
Transverse colon	1	0	
Ascending + transverse colon	1	0	

Hinchey classification, 56 patients were diagnosed with noncomplicated acute diverticulitis (Hinchey 0,1a) and 26 patients were diagnosed with complicated diverticulitis (Hinchey 1b, 2,3,4) based on CT findings and clinical findings (Table 2).

In mean age and gender distribution between complicated acute diverticulitis patients and noncomplicated acute diverticulitis patients had no difference as significantly (Table 2). In the distribution of the location of diverticulitis in the colon between the groups had no difference as statistically significant (Table 2).

Duration of hospital stay was longer in CAD than in the SAD (*p* < *0.001*) as statistically significantly (Table 2). Rate of surgical procedures and percutaneous interventions was statistically significantly higher in CAD than SAD (*p:0.040*) (Table 2). Surgery was performed on 4 patients; primary colon repair was performed on 3 patients and colon resection and anastomosis were performed on 1 patient.

Blood values and calculated inflammatory parameters, WBC (white blood cell), CRP, NLR, SII dNLR and SIRI were higher in CAD than SAD as statistically significant (Table 3). PLR and MLR rates were higher in CAD but no statistical difference was found.

**Table 3:** Differences of WBC, CRP, NLR, dNLR, SII, SIRI, PLR and MLR values between groups

	SAD	CAD	p*
WBC	8,868	13,610	0.000*
CRP	55,540	104,277	0.016*
NLR	3,726	6,637	0.022*
dNLR	4,245	7,515	0.015*
SII	838,074	1888,476	0.013*
SIRI	2,976	5,766	0.007*
PLR	134,749	162,900	0.166
MLR	0.426	0.5091	0.222

There was a correlation between Hinchey classification and WBC, CRP, NLR, dNLR, SII, and SIRI parameters and among these parameters as a very high positive according to Spearman’s correlation analysis (Table 4).

**Table 4:** Spearman correlation test results

Correlations									
			HINCHEY	wbc	CRP	nlr	d- nlr	SII	SIRI
Spearman's rho	HINCHEY	Correlation Coefficient	1.000	0.496 ^**^	0.363 ^**^	0.342 ^**^	0.384 ^**^	0.386 ^**^	0.414 ^**^
		Sig. (2-tailed)		0.000	0.001	0.002	0.000	0.000	0.000
	wbc	Correlation Coefficient	0.496 ^**^	1.000	0.468 ^**^	0.622 ^**^	0.633 ^**^	0.709 ^**^	0.722 ^**^
		Sig. (2-tailed)	0.000		0.000	0.000	0.000	0.000	0.000
	CRP	Correlation Coefficient	0.363 ^**^	0.468 ^**^	1.000	0.527 ^**^	0.549 ^**^	0.453 ^**^	0.594 ^**^
		Sig. (2-tailed)	0.001	0.000		0.000	0.000	0.000	0.000
	nlr	Correlation Coefficient	0.342 ^**^	0.622 ^**^	0.527 ^**^	1.000	0.995 ^**^	0.906 ^**^	0.939 ^**^
		Sig. (2-tailed)	0.002	0.000	0.000		0.000	0.000	0.000
	d- nlr	Correlation Coefficient	0.384 ^**^	0.633 ^**^	0.549 ^**^	0.995 ^**^	1.000	0.888 ^**^	0.939 ^**^
		Sig. (2-tailed)	0.000	0.000	0.000	0.000		0.000	0.000
	SII	Correlation Coefficient	.386 ^**^	0.709 ^**^	0.453 ^**^	0.906 ^**^	0.888 ^**^	1.000	0.865 ^**^
		Sig. (2-tailed)	0.000	,000	0.000	0.000	0.000		0.000
	SIRI	Correlation Coefficient	0.414 ^**^	0.722 ^**^	0.594 ^**^	0.939 ^**^	0.939 ^**^	0.865 ^**^	1.000
		Sig. (2-tailed)	0.000	0.000	0.000	0.000	0.000	0.000	

^**^ Correlation is significant at the 0.01 level (2-tailed)

^*^ Correlation is significant at the 0.05 level (2-tailed)

**Fig. 1:**
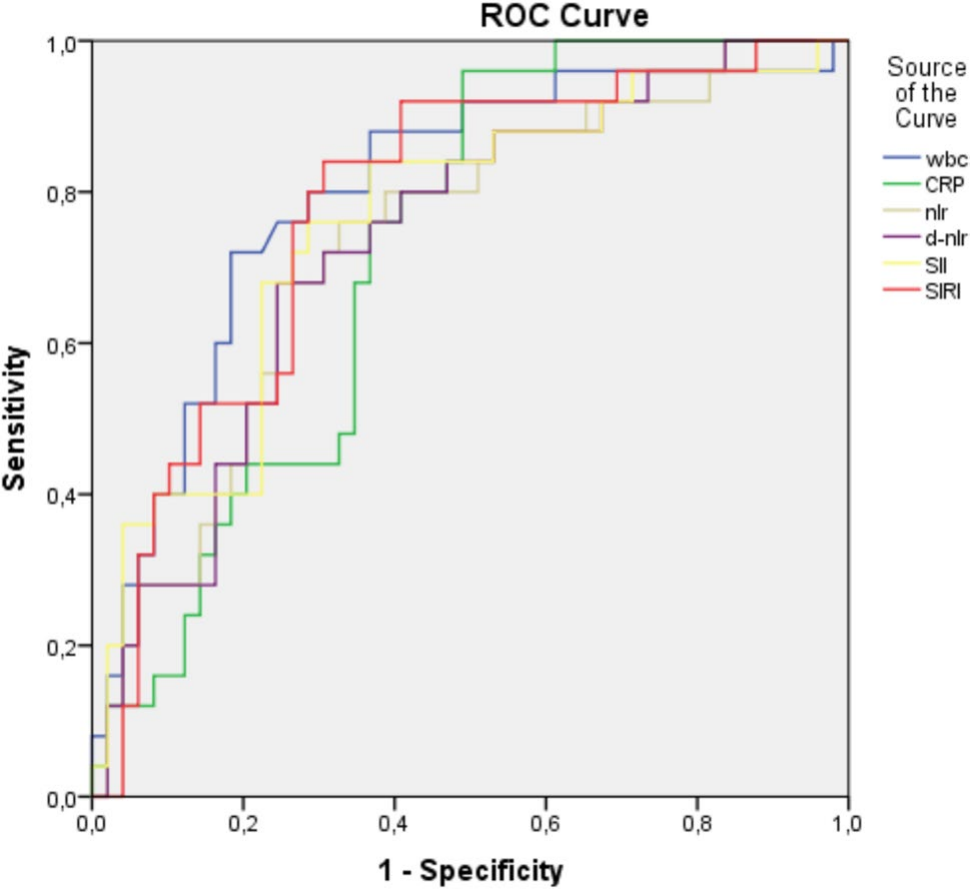
ROC analysis for WBC, CRP, NLR, dNLR, SII, SIRI in the distinction between SAD and CAD

Analysis of ROC was performed to evaluate the diagnostic accuracy of CRP, WBC NLR, dNLR, SIRI and SII values used in the detection of complicated diverticulitis patients and to establish cut-off value (Fig. 1).

AUC was established to determine the prognostic effect. Highest AUC value was found in WBC followed by SIRI, SII, NLR, and dNLR, respectively (Table 5).

**Table 5:** ROC analysis for WBC, CRP, NLR, dNLR, SII, and SIRI values in predicting CAD

	Cut Off	AUC	SENSITIVITY	SPECIFICATION	*p*
NLR	3.35	0.740(0.624–0.854)	73.1%	67.3%	0.001
dNLR	4.63	0.739(0.625–0.853)	68.0%	74.5%	0.001
SII	907.44	0.767(0.654–0.880)	73.1%	72.7%	0.000
SIRI	2.42	0.786(0.681–0.892)	80.8%	69.1%	0.000
CRP	15.25	0.722(0.608–0.835)	96.0%	51.0%	0.002
WBC	11.16	0.807(0.703–0.910)	73.1%	81.8%	0.000

Cut-off points were established with the Youden index (Table 5). Sensitivity and specificity values were established with cut-off points.

## Discussion

Diverticular disease is a bowel pathology that is more common in older ages. Just about in 10–25% of diverticulosis patients develop acute diverticulitis [21]. The prognosis varies according to the seriousness of the disease. Mild, uncomplicated diverticulitis can be treated on stand. For complicated patients may be necessary to use minimal invasive treatment or even surgery. Determining the severity of acute diverticulitis is important for establishing the time of hospital stay, surgical or percutaneous needs of the patients. Abdominal tomography can be evaluated along with WBC and CRP values to determine seriousness of the disease and seriousness of the disease according to Hinchey’s classification. Abdominal tomography may not be available in every center. Abdominal tomography gives disease severity but patients are exposed to radiation during this procedure. For the reasons mentioned, simpler and cheaper methods are being investigated in centers without tomography or to reduce radiation exposure, as in diverticulitis patients.

In this retrospective study, data obtained from blood hemogram values in acute diverticulitis patients were used for establishing the severity of diverticulitis disease using simple mathematical calculations. The relationship of these data was examined with the Hinchey classification and it was concluded that using them to determine the severity in patients with acute diverticulitis could be useful. This study is the only in the literature where SII, SIRI dNLR, NLR, CRP, and WBC were used together in patients with acute diverticulitis.

In this study, the number of male patients were fourty-one and number of female patients were fourty-one. Of the 82 patients, 31.7% [26] had acute complicated diverticulitis. This was slightly higher than the study of Bharucha A et al. In Barucha’s study, there were 3222 patients and found that 12% (*n* = 386) were CAD [22].

Spearman correlation test, SII, SIRI dNLR, NLR, CRP, WBC values showed a very high correlation with the Hinchey classification as positive (Table 4).

The mean WBC values were higher in CADs as statistically significantly (13.6 × 10^9^/L) than in patients with SAD (8.68 × 10^9^/L) (*p: 0.000*). It is thought that measuring the difference between each hemogram value rather than measuring them separately is more accurate in predicting the results of some clinical pathologies [23]. In previous studies on WBC, CRP, NLR, and PLR, it was found that complicated diverticulitis and surgical intervention were associated with high NLR, PLR values. [24–27]. In this study, NLR values were lower in SADs as statistically significantly (3.726) than in patients with CAD (6.637) (*p: 0.022*). In ROC analysis, 73.1% sensitivity and 67.3% specificity values were found for a point of 3.35 (*p*: 0.001).

Tan et al. showed a strong correlation between the severity of acute diverticulitis and high CRP levels. [28]. CRP values were higher in CADs than SAD as statistically significantly (*p*: 0.016), and there was a correlation between Hinchey classification and CRP in the Spearman correlation test as very high positive (*p: 0.001*).

Systemic immune-inflammatory index (SII) was founded as a trustworthy marker for malignancy patients as prognostic evaluation and intra-abdominal inflammatory studies have also been conducted in pathologies [29, 30]. We found that the SII value was higher in CADs than SADs as statistically significantly in our study (*p*: 0.013). ROC analysis performed by us determined a cut-off value, 73.1% sensitivity and 72.7% specificity rates were detected for 907.44 value.

SIRI has been investigated in some studies, for patients with malignancy, to predict prognosis [31]. No studies on SIRI values in patients with acute diverticulitis were found in the literature. Our study is unique in the literature in evaluating SIRI in acute diverticulitis patients. In this study, SIRI mean value was higher in CADs (5.766) than SADs (2.976) statistically significantly (*p: 0.007*). In the Spearman correlation test, it was shown to have a very high correlation with the Hinchey classification as positive (*p: 0.000*). In the ROC analysis we conducted to determine a cut-off point, a sensitivity of 80.8% and a specificity of 69.1% were found for 2.42 value (*p*: 0.000).

Some clinical studies, derived NLR (dNLR) has been studied instead of NLR. dNLR has been studied in breast cancer. The effect of predicting prognosis was examined [32, 33]. dNLR is calculated by dividing absolute neutrophil count by derived lymphocyte count (absolute leukocyte count-neutrophil count)]. In the literature, there are publications stating that dNLR is an important indicator for acute diverticulitis. There is no study on its use in determining the prognosis. With this study, we showed that dNLR and SIRI values are useful in determining the prognosis of acute diverticulitis. This is the only study in the literature that evaluates its use in determining prognosis. In our study, the dNLR value was higher in CADs than in SADs as statistically significantly (*p*: 0.015). In ROC analysis, sensitivity was determined as 68.0% and specificity was determined as 74.5% for a cut-off point of 4.63 (*p*:0.001).

Butyrylcholinesterase (BChE) is a type of cholinesterase enzyme. BChE is being studied as a research topic for colon inflammation or post-surgical infections. Specific agents like this could be used as another study topic in patients with diverticulitis [34].

Study has some limitations. First that it is retrospective, and the sample size is not very high. In this study, strengths are that this is the only diverticulitis study in the literature that evaluates SII, SIRI dNLR, NLR, CRP, and WBC values together and the only diverticulitis study in the literature that uses SIRI, and dNLR values.

The separation of patients coming to the hospital emergency services due to acute diverticulitis into complicated and uncomplicated determines whether the patients will be admitted to the hospital and whether they will need surgery or minimally invasive surgery. The use of tomography together with the blood parameters used to make this distinction is detrimental both in terms of cost and radiation given to the patient. SII, SIRI dNLR, NLR, CRP, and WBC values can be studied and used easily and less costly. In this way, it will prevent unnecessary tomography and allow us to make faster treatment planning for complicated cases. We believe that supporting these data with larger randomized controlled prospective studies will provide benefits in terms of using the data more clearly and protecting from cost and radiation.

## Conclusion

In acute diverticulitis patients, there are very high correlation SII, SIRI, dNLR, NLR, CRP, and WBC values with the Hinchey classification in distinguishing complicated and uncomplicated acute diverticulitis. (*p* < *0.01*). These data were higher in CAD patients than in SAD patients as statistically significantly. Use of these data can both prevent unnecessary radiation in patients suspected of acute diverticulitis by reducing unnecessary tomography scans and it may be a logical choice to predict the prognosis of patients at low cost.
